# Structuring and validating a cost-effectiveness model of primary asthma prevention amongst children

**DOI:** 10.1186/1471-2288-11-150

**Published:** 2011-11-09

**Authors:** G Feljandro P Ramos, Sandra Kuiper, Edward Dompeling, Antoinette DI van Asselt, Wim JC de Grauw, J André Knottnerus, Onno CP van Schayck, Tjard RJ Schermer, Johan L Severens

**Affiliations:** 1 Department of General Practice, CAPHRI School for Public Health and Primary Care, Maastricht University, Maastricht, the Netherlands; 2 Department of Paediatric Pulmonology, Maastricht University Medical Centre, Maastricht, the Netherlands; 3 Department of Clinical Epidemiology and Medical Technology Assessment, Maastricht University Medical Centre, Maastricht, the Netherlands; 4 Department of Primary and Community Care, Radboud University Nijmegen Medical Centre, Nijmegen, the Netherlands; 5 Department of Health, Organisation, and Policy Economics, CAPHRI School for Public Health and Primary Care, Maastricht University, Maastricht, the Netherlands; 6 Institute of Health Policy and Management, Erasmus University, Rotterdam, the Netherlands

## Abstract

**Background:**

Given the rising number of asthma cases and the increasing costs of health care, prevention may be the best cure. Decisions regarding the implementation of prevention programmes in general and choosing between unifaceted and multifaceted strategies in particular are urgently needed. Existing trials on the primary prevention of asthma are, however, insufficient on their own to inform the decision of stakeholders regarding the cost-effectiveness of such prevention strategies. Decision analytic modelling synthesises available data for the cost-effectiveness evaluation of strategies in an explicit manner. Published reports on model development should provide the detail and transparency required to increase the acceptability of cost-effectiveness modelling. But, detail on the explicit steps and the involvement of experts in structuring a model is often unevenly reported. In this paper, we describe a procedure to structure and validate a model for the primary prevention of asthma in children.

**Methods:**

An expert panel was convened for round-table discussions to frame the cost-effectiveness research question and to select and structure a model. The model's structural validity, which indicates how well a model reflects the reality, was determined through descriptive and parallel validation. Descriptive validation was performed with the experts. Parallel validation qualitatively compared similarity between other published models with different decision problems.

**Results:**

The multidisciplinary input of experts helped to develop a decision-tree structure which compares the current situation with screening and prevention. The prevention was further divided between multifaceted and unifaceted approaches to analyse the differences. The clinical outcome was diagnosis of asthma. No similar model was found in the literature discussing the same decision problem. Structural validity in terms of descriptive validity was achieved with the experts and was supported by parallel validation.

**Conclusions:**

A decision-tree model developed with experts in round-table discussions benefits from a systematic and transparent approach and the multidisciplinary contributions of the experts. Parallel validation provides a feasible alternative to validating novel models. The process of structuring and validating a model presented in this paper could be a useful guide to increase transparency, credibility, and acceptability of (future, novel) models when experts are involved.

## Background

Asthma affects more people [1,2] and costs more to manage than ever before [3-6]. Given limited resources for competing needs, prevention of asthma could pay off. Currently, primary prevention programmes on asthma generally differ in the approach to reduce or avoid exposure to one (unifaceted, e.g. an anti-house dust mite [HDM] measure) or more environmental factors (multifaceted, e.g. exclusive breastfeeding until six months and an anti-HDM measure) [7].

The decision to ultimately implement asthma prevention programmes, let alone choose between a unifaceted and a multifaceted approach, is not made easier with favourable [8,9] and unfavourable [10,11] reports on the effectiveness of prevention. Differences in the conclusions could be due to factors such as the essential difference between the approaches (unifaceted versus multifaceted), the population (open versus captive), "room for improvement" (pre-existing high-levels of allergen avoidance or adapted smoking behaviour), and even the selected outcome measures or (asthma) definitions (e.g. asthma-related symptoms, physician-diagnosis). However, the meta-analyses by van Schayck and colleagues [12,13] show that multifaceted programmes significantly reduce the risk for asthma compared to unifaceted programmes.

Although several studies have been performed on primary prevention of asthma amongst children, none could singly determine the cost-effectiveness of primary prevention [8-11,14-18]. Specifically, there is no known study that directly compares the various alternatives in primary asthma prevention [12,13]. Ideally, it could be studied in a clinical trial or, alternatively, using a model which synthesises the evidence from all available sources and levels of evidentiary quality [19,20]. Such a model could estimate the uncertainty of decisions and cost associated with asthma given imperfect, current data in an explicit and transparent manner [19].

A permutation of models is possible depending on various considerations but, despite the pervasive use of models in economic evaluation, there is still wariness about their appropriateness and validity [19,21]. However, movement towards uniformity through good practice guidelines [21-24] have helped to allay such wariness. Still, there is a need for more transparency and explicitness in modelling practice [25]. Already, this is being met in clinical effectiveness research by the increasing practice of reporting study protocols separate from the trial results [26,27], which reduces publication bias and increases transparency and credibility. In this paper, we describe the design and validation of the structure of a decision analytic model which compares multifaceted and unifaceted programmes of primary prevention of asthma in children versus the current situation (i.e. unstructured advice on avoiding allergen sensitization and symptomatic treatment).

## Methods

### Expert panel sessions and model structuring

Consultation with experts, just as personal experience or a review of the literature, may provide the background information needed when developing a model [28]. For this paper, experts were consulted to provide biomedical insight into the natural course of asthma and its care in the Netherlands. We developed a protocol (Additional File 1) on conducting the expert panel held as round-table sessions [29]. The protocol identified relevant inputs and expected outcomes of each session, including the guiding questions to structure the discussions. Following a review of relevant health technology assessment (HTA) literature [28-30], the protocol consists of the following elements:

1. Description of the study's background, motivation, and objectives including the role of experts in the modelling process;

2. Identification of criteria for selecting experts;

3. Identification and inclusion of experts;

4. Identification of the number and purpose of the expert sessions;

5. Description of the structure of the sessions in terms of knowledge, process events, and expected session outcomes;

6. Description of the techniques to referee between experts, including the feedback communication with the experts; and

7. Documentation of the results.

The foremost criterion for expert selection was the extent of their expertise and experience with asthma and its prevention in children and/or research experience in the field of efficacy and cost-effectiveness [29]. This was discussed within the research group which resulted in nominations for candidate experts. Once identified and contacted, the experts' willingness to participate and ability to attend the sessions [29] were also considered. The experts received copies of the protocol for their guidance to the process. Initially, only two sessions were planned but because of logistical issues as well as significant changes in the model structure after consulting the research group, another session was added. Consensus was not formalised as strict unanimity on discussion points because of the explorative nature of the round-table discussions [29].

The expert panel sessions were conducted in 2010 at Maastricht University, the Netherlands. Eight expert nominees were contacted but the final combination of experts were two from general practice and (paediatric) pulmonology and one each from epidemiology and HTA. During the first session, the HTA expert joined in by telephone and thereafter kept contact through e-mail. Because of unavailability, one pulmonologist and one general practitioner (GP) dropped out after the first and second sessions, respectively.

The first session was meant to frame the decision problem and generate assumptions needed to determine the structure of the model. Input to this session was an overview of the research line as background for the decision problem and purpose of the model. The experts were asked about other aspects of the decision framework covering the natural history of the disease, gold standards of measure, possible interaction and recursion of disease states, as well as appropriate comparators. Since time horizon, population, and perspective were already naturally defined by the existing clinical studies from the PREVention of ASthma amongst Children (PREVASC) research line [14-18], these elements were not solicited from the experts. The first session resulted in a set of assumptions and a decision framework.

After the first expert panel session, the literature was systematically searched for existing models that could be adapted for the current decision problem [31]. When a new model was required, the type was chosen using guidelines and recommendations in the literature [21-24]. Consideration was given to the purpose (single-use vs. programmatic), level of simulation (individual vs. cohort), relationship between individuals (independent vs. interacting), and frequency of outcomes/health states (recursive vs. non-recursive). The model structure was drawn based on the type of model, the decision framework and assumptions, and published recommendations [32].

The last sessions were meant to refine the model structure and determine its descriptive validity. For the second session, the input was an overview of the modelling process and the general types of models, to provide some information to the experts with limited exposure to economic modelling. It was only given at that time so that the generation of assumptions and the framing of the decision problem during the first session would not be restricted by bias for one model or another. During this second session, the model structure was scrutinised on how well it reflected the set of assumptions and decision framework from the first expert panel session. The outcome at this stage was a list of comments for improving the correspondence of the model structure with the set of assumptions and decision framework. On the third and last expert panel session, the input was the latest model structure which was again evaluated. After satisfying the descriptive validity, the model was finalised.

### Validating the model

Descriptive validity (i.e. whether the essential characteristics of reality is captured in the model [33-35]) was determined during the second and third expert panel sessions. This validation was carried out with the experts' review of how the model structure was able to incorporate the assumptions about the development and current care of the disease into a simplified structure. Specifically, it was determined during the sessions whether the model was parsimonious and sufficient to answer the decision problem [36]. This was an iterative process with the experts after the initial session.

**Figure 1:**
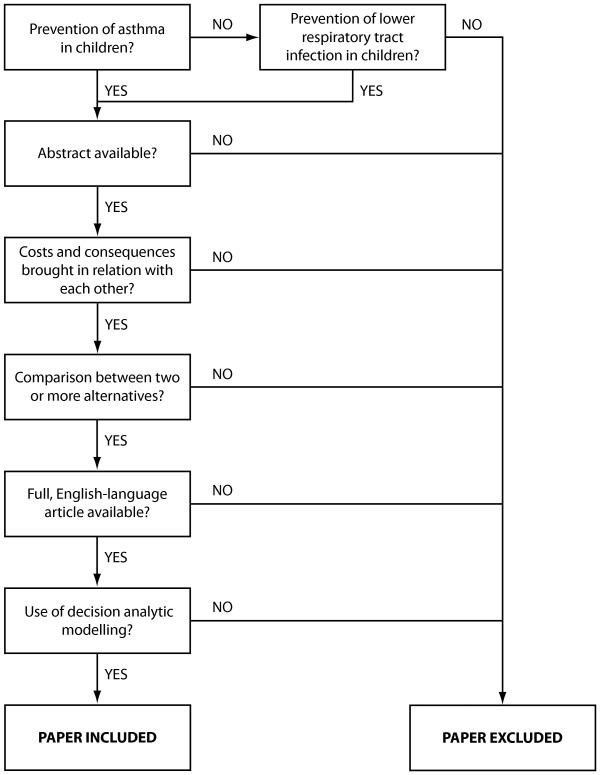
**Selection of economic evaluations for the modified convergent validation**. This flow chart shows the process of selecting the studies which would be included in the analysis for parallel validation.

In the absence of comparable models that evaluate allergen-avoidance measures in the primary prevention of asthma in children, we focused instead on the parallelisms (i.e. similarities) between characteristics of the model structure, namely: model type, time horizon, perspective, population, intervention, comparator, and health states/effect measures [37]. The other characteristics are related to the natural history of a disease and its treatment which are currently important determinants in the chain of choices that lead to the selection of the model structure [37]. Aside from including studies on the secondary and tertiary prevention of asthma (i.e. preventing allergic sensitisation from progressing to asthma or preventing the exacerbations of established asthma [1]) we proceeded with this "parallel validation" by increasing the scope to studies on lower respiratory tract infections. These infections in infants, particularly with the respiratory syncytial virus, have been associated with the development of asthma later on [38]. To test for the parallelisms, we first performed a systematic search of the literature to identify the relevant studies. The search was conducted in the PubMed databases based on an extensive search strategy for economic evaluations [39,40]. The database was searched until 08 September 2010 and was restricted to English-language records of titles and abstracts of evaluations that used decision-analytic modelling. Only evaluations of at least two alternatives on asthma prevention which relate costs to consequences [41] were included in the review. Following the screening process (Figure 1), the results were reviewed by one of the co-authors and any uncertainty was resolved by direct discussion. Technical or internal validity of the calculations and consistency of formulae and parameter inputs with the assumptions were checked.

### Model operation

**Figure 2:**
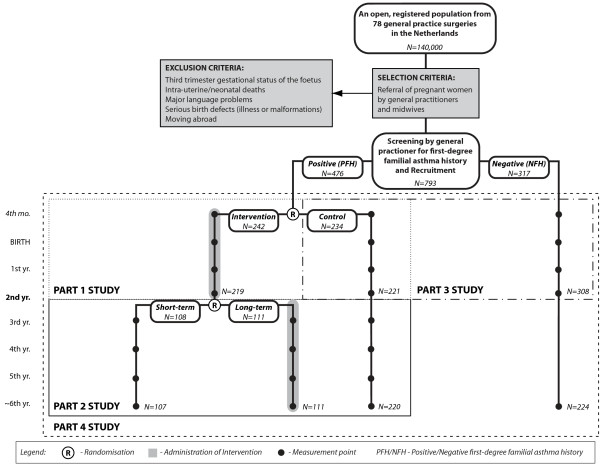
**The PREVASC Research Line**. Schematic flow of the participants in the different studies of the PREVASC research line beginning with the risk-screening of the sample population indicates that data synthesis through modelling is necessary to determine the value of multifaceted primary asthma prevention amongst children. Measurement around the age of six for subjects with negative history is part of the current (Phase 4) study.

Once the decision problem was framed and the model's structure visualised, its operational feasibility or technical validity [35] was tested by populating it with data from the PREVASC studies. Detailed description of the PREVASC programme (Figure 2) can be found elsewhere [14-18]. Children were included in the studies based on risk for first-degree familial asthma history (i.e. positive or negative, PFH or NFH respectively) [14,15]. Phase 1 and 2 studies investigated the clinical effects of multifaceted primary asthma prevention at age two and six years, respectively. It was found that over the short or long term, there were no significant reductions of asthma diagnosis between intervention and control groups [16]. However, a subgroup of the PREVASC children seemed to benefit from the prevention programme [14]. Phase 3 was a natural history study which found that there were more respiratory-tract morbidities and atopy in PFH than NFH children in the first two years of life [19,20].

Branch probabilities were calculated as the ratio of one situation over its sum with the complement possibilities. A diagnostic algorithm (Table 1) was applied to determine asthma status based on inhaled corticosteroid (ICS) use, wheezing three months prior to the lung function measurements, and the measurements on reversibility and hyperreactivity. Because the original PREVASC clinical studies did not explicitly study the effects of unifaceted prevention, this had to be derived from the clinical data. A per-protocol analysis of compliance was used to reclassify PFH participants into multifaceted, unifaceted, or control. The current situation was a combination of the PFH control and NFH participants weighted by the respective ratios of 20% and 80% found in the general population [42].

**Table 1:** Algorithm for objective asthma diagnosis of children

Inhaled corticosteroid treatment 3 months prior to lung function assessment	Symptom/ Complaint	Reversibility	Hyperreactivity	Asthma
-	+	+	Not Needed	+
	+	-	+	+
	+	-	-	-
	-	+	+	+
	-	+	-	-
	-	-	Not Needed	-

+	Not Needed	+	+	+
	Not Needed	+	-	+
	Not Needed	-	+	+
	Not Needed	-	-	Sensitivity Analysis

Direct medical as well as intervention costs were assessed and valued using standardised unit prices for the Netherlands indexed using 2009 figures [43]. These direct medical costs include GP and specialist consultations for asthma-related complaints, any related hospital admissions, the diagnostic tests (i.e. chest x-ray and radioimmunoassay test for allergens), and medication use. For those who received intervention, costs on research nurse visits, the use of house dust mite-impermeable materials for the beds of participants and parents, hypoallergenic formula feeding, and education materials in the form of brochures were included. The model outcome was the incremental cost-effectiveness ratio which was assessed for various threshold values (€0 - €70,000 per asthma case avoided).

## Results

### Expert panel and sessions

**Figure 3:**
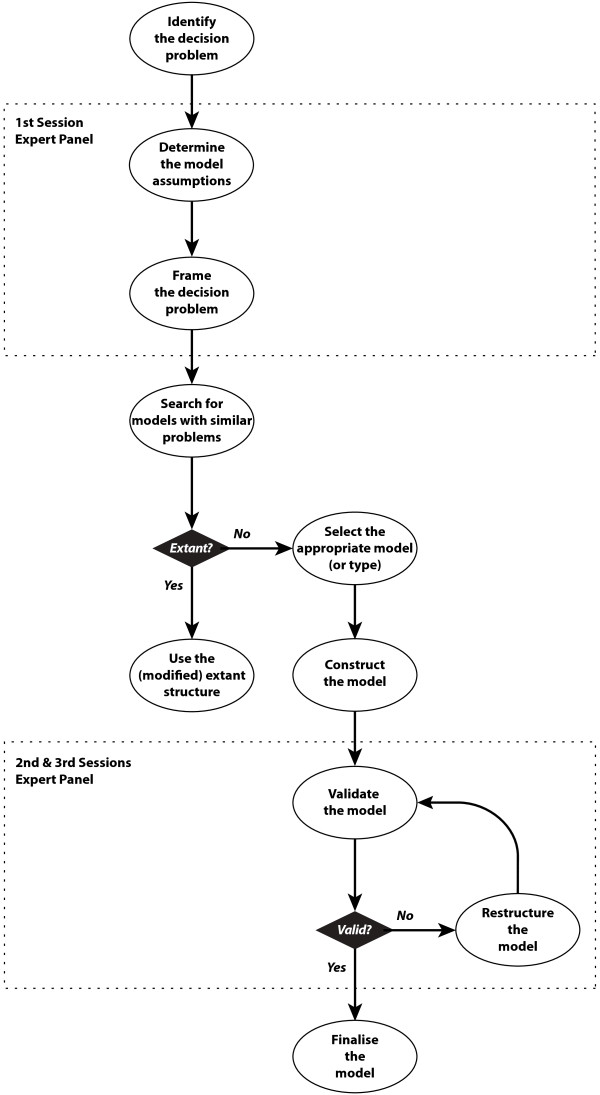
**Expert input in the model development**. Flowchart showing the influence of experts regarding the design and validation of the structure of the model to determine the economic value of primary prevention in asthma.

Clinical insight into the natural course of asthma, diagnosis, as well as current care and alternatives were provided by experts from the first three fields. The HTA expert provided insight into important economic considerations such as how costs and consequences can be incorporated in the comparison of alternatives. The involvement of the experts in the development of the model structure is illustrated in Figure 3.

### Decision framework and assumptions for the model structure

Discussion amongst the experts resulted in a decision framework (Table 2) that determined the most appropriate model. This framework was incumbent on a number of expert-derived assumptions largely drawn from the biomedical model of asthma. The experts considered asthma to be more than the sum of genetic expression and environmental influences that might be best addressed by multifaceted programmes. They estimated clinicians would see a larger number of allergic children with transient wheezing than the classic asthma presentation of persistent wheezing combined with shortness of breath and (nocturnal) coughing. Most asthma cases that would be seen, they thought, would be the allergic variant typically seen in children exposed to common allergens for the first time. The experts argued that the processes that lead to asthma may have begun with allergic sensitisation in utero. They cited that the current paradigm of asthma prevention in the Netherlands consists of unstructured pre- and post-natal advice to mothers and their families regarding exposing the child to environmental tobacco smoke and air-borne particulates for children in general and pharmacotherapy for those with symptoms. The experts assumed that any alternative intervention must also be instituted during gestation and after birth to prevent asthma. For any prevention programme, a reasonable screening strategy is necessary to identify the recipients who are most at risk and stand to gain the most from the programme. It was thought that this would be best done through first-degree familial asthma history.

**Table 2:** Decision problem framework for the primary prevention of asthma in children

Framework Aspect	Detail
Objective(s)	To reduce the incidence of asthma diagnosis in children To reduce the costs associated with asthma health care To determine the value of first-degree familial asthma history as a risk indicator
Audience/Stakeholder(s)	Primary care givers (primary care or welfare centre physicians, midwives, and general practice/medical assistants) and Medical specialists (pulmonology and paediatrics)
Perspective	Healthcare system
Analytic time horizon	Up to six years after birth
Intervention(s)	Multifaceted primary prevention Unifaceted primary prevention
Comparator	Usual care
Target population	Unborn child
Effect outcome(s)	Objective diagnosis of asthma Secondary: Functional status; Serum IgE levels as index of exposure to allergens from house dust mites, cats, and dogs
Cost(s)	Direct health care costs; Intervention costs
Economic evaluation	Cost-effectiveness

Although differentiating between phenotypes was recognised as invaluable in refining asthma diagnosis, it was contended that the diagnosis of asthma alone was sufficient and necessary to determine the value of primary prevention at the health care level. Even if diagnosis were to be established at age six years, it was assumed that the pathological processes may have begun long before diagnosis. Furthermore, these processes may continue even in the absence of clinical signs and symptoms at later years. However, they noted that diagnosis of asthma prior to age six years is difficult or changes over time. Patients would be seen when the flare of symptoms as well as the pathologic process are subsiding which may not give a very accurate picture of the disease. At a later age, clinical remission may occur during pubescence or it may recur during the adult years. The difficulty, they said, is compounded by a lack of gold standard for its diagnosis. Around age six years, large groups of children who may have asthma are symptom-free but physiologic changes such as exaggerated inflammatory reactions and airway remodelling may already be occurring which could be detected using objective measurements. According to the experts, bronchioalveolar lavage and biopsies are the most reliable tests for airway inflammation. But since the procedures are also invasive and difficult to perform in practice, those were considered primarily research more than routine clinical tools. In contrast, the experts said that spirometry is common in the general practice setting and suitable in documenting abnormal lung function described by reversibility and hyperresponsiveness of the bronchial tubes. Combining objective lung function tests with symptomatology seemed to them to be the best approach to asthma diagnosis to date. However, they said that since symptomatic children in the Netherlands receive asthma medication, particularly ICS, an objective diagnosis at age six years may be contaminated. It was then assumed that children who received ICS therapy three months prior to the tests are asthmatic when either test of reversibility or hyperreactivity is positive. When both tests are negative, diagnosis could go either way. Amongst children who did not receive ICS therapy, positive hyperreactivity was assumed to signify presence of asthma and negative hyperreactivity meant absence of asthma. It was also assumed that evaluating the costs and consequences over a time horizon of six years after birth would be sufficient since this is the age when lung function tests would be first reliable in children.

### Model structure

**Figure 4:**
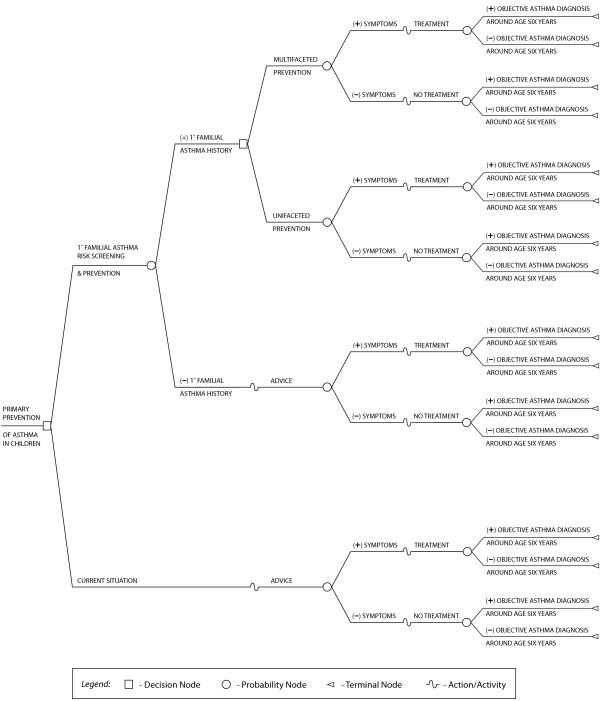
**The PREVASC Decision Tree**. Structure of the decision analytic model to assess the cost-effectiveness of primary prevention of asthma. The tree compares current situation without primary prevention with the alternative of primary prevention of asthma amongst children (first decision node) as well as whether multifaceted or unifaceted primary prevention approaches is more cost-effective (second decision node).

A search of the literature for economic models of primary asthma prevention in children based on the expert-developed decision framework came up empty-handed (Table 3). This meant that a new model (Figure 4) had to be developed which began with the problem or situation of administering to children a primary prevention programme on asthma. Based on the expert sessions, it was determined that the decision tree structure suited the decision framework and assumptions. Identifying the target population for the intervention on the basis of risk became the first node for the intervention arm of the decision tree. It was assumed that NFH children would receive current care [44] whereas PFH children would be given the intervention either as a unifaceted or multifaceted prevention depicted by the second decision node coming from the PFH branch. A child would receive unifaceted prevention when only one measure against air- or food-borne allergens is employed and multifaceted when at least two measures against both triggers are used. It was also assumed that children could not shift between these two prevention categories. The model resulted in three situations of care: prevention with unifaceted or multifaceted approach and current situation care. For these situations, children were assumed to either remain healthy or manifest some asthma signs and symptoms. Consequent upon the clinical picture, the children may or may not receive pharmacotherapy as required. The primary outcome is either a positive or a negative objective diagnosis of asthma, which relies on reversibility and hyperreactivity tests and symptomatology.

**Table 3:** Results for the systematic review for decision analytic models on asthma prevention

Step	Search String	Hits
#1	("Costs and Cost Analysis/classification"[MeSH] OR "Costs and Cost Analysis/economics"[MeSH] OR "Costs and Cost Analysis/education"[MeSH] OR "Costs and Cost Analysis/ethics"[MeSH] OR "Costs and Cost Analysis/history"[MeSH] OR "Costs and Cost Analysis/legislation and jurisprudence"[MeSH] OR "Costs and Cost Analysis/methods"[MeSH] OR "Costs and Cost Analysis/organization and administration"[MeSH] OR "Costs and Cost Analysis/standards"[MeSH] OR "Costs and Cost Analysis/statistics and numerical data"[MeSH] OR "Costs and Cost Analysis/trends"[MeSH] OR "Costs and Cost Analysis/utilization"[MeSH])	26349
#2	Cost-effective*[Title/Abstract]	54952
#3	#1 OR #2	78623
#4	Journal Article[ptyp] AND English[lang]	14875628
#5	"Animals"[MeSH:NoExp]	4570915
#6	"Humans"[MeSH:NoExp]	1125512
#7	#5 NOT (#5 AND #6)	3397345
#8	(#3 AND #4) NOT #7	67344
#9	Asthma*[Title/Abstract] or "Asthma/prevention and control"[MeSH]	99545
#10	#8 AND #9	586
#11	#10 NOT Review[Ptyp]	406
#12	"primary prevention"[Title/Abstract] or "secondary prevention"[Title/Abstract]	16821
#13	#11 AND #12	2
#14	#10 AND model[Title/Abstract]	62

### Validation of the model structure

The model structure was revised and refined in the last two sessions with the experts who were asked to consider the intuitiveness of the model structure in reflecting the decision framework and the assumptions. The review was at the same time a process of descriptively validating the model structure. Concerning the parallel validity with models in the literature, the strategy and resulting hits are shown in Table 3. In reviewing the titles and abstracts, none of the 406 search results referred to a trial-based economic evaluation of primary or secondary asthma prevention in children which used a non-pharmacological approach. When the survey focused on models, there were 62 hits, of which only seven were studies on the pharmacological prevention of asthma or lower respiratory tract infection in children. After a critical appraisal, two studies were further excluded because they did not use decision analytic models in the economic evaluation. Of the remaining five studies (Table 4), three [45-47] used decision-tree models and two [48,49] used Markov models. Two studies focused on asthma prevention [46,49] and others on RSV infection [45,47,48]. Omnes and colleagues [46] reported a model which has the most characteristics similar to ours in terms of model structure, time horizon, perspective, population, comparator, and effects. The intervention was, however, not a perfect correspondence which is not surprising given the lack of comparable models with our decision framework. In short, their study supports the structural validity of our model and decision problem's most salient concerns and so validates in a way the structure of the model presented here through parallelisms.

**Table 4:** Important characteristics of studies for parallel validation

Study	Model	Time Horizon	Perspective	Population	Intervention	Comparator	Effects
Nuijten [45]	Decision tree	Lifetime	UK National Health Service and society	Pre-term infants and children with BPD	Palivizumab	No prophylaxis	Number of RSV hospitalizations avoided
Omnes [46]	Decision tree	7 years (6 years-adults)	French Social Security	Children and adults	Specific immunotherapy (injectable and sublingual)	Current symptomatic treatment	Proportions of individuals with rhinitis or allergic asthma
Resch [47]	Decision tree	Lifetime	Austrian third party payer and society	Infants born premature or with BPD, and children with congenital heart disease	Palivizumab	No prophylaxis	Life years gained and QALY gained
ElHassan [48]	Markov (with and without increased asthma risk due to RSV infection)	1 year (no-risk); 8-10 years (with risk)	US society	Premature infants	Palivizumab	No prophylaxis	QALY gained
Brüggenjürgen [49]	Markov model	15 years	German third party payer and society	Children (6 to 12 years), adolescents (13 to 18 years), and adults (19 to 65 years)	Specific immunotherapy (subcutaneous) and symptom treatment	Symptom treatment	QALY gained

QALY-quality-adjusted life year.

BPD = bronchopulmonary dysplasia; RSV = respiratory syncytial virus; QALY = quality-adjusted life years

### Model operation

For this demonstration of the model's operation and internal validity, we have used solely the data from the PREVASC studies. Only cases with relevant diagnostic data were included in the modelling which whittled down the possible 443 PFH cases in the intervention and control group to 324 cases. Their compliance with at least two, one, or none of the measures against exposure to airborne and food allergens was the basis for respectively regrouping them into multifaceted (N = 259), unifaceted (N = 53), and control (N = 12). Branch probabilities were calculated and folded back for the expected outcomes and costs. Between the prevention approaches, the multifaceted had a higher costs but also a higher expected probability of asthma cases avoided than the unifaceted approach (€539.49 vs. €445.19 for costs and 0.93191 vs. 0.92610 for probability of asthma avoidance, respectively). This means that the multifaceted approach costs around €16,000 more to avoid one asthma case than in the unifaceted approach. However, when compared with the current situation, which has the lowest cost and highest probability (€302.30 and 0.96494), the two prevention approaches were strongly dominated. Even for various cost-effectiveness thresholds, the current situation dominated the two approaches.

## Discussion

### The model structure

Use of models in health economic evaluation has increased over the years and most often for one of two things [50]. First, when the relevant clinical trial has not (yet) been conducted or economic data is missing or incomplete. Second, when the clinical trial used intermediate outcomes or when the follow-up period is short whilst the decision problem is concerned with endpoints long into the future. Though modelling allows decision makers to be critical and realistic about alternatives, to be conscious and clear of valorisation, and to reveal the relationships between inputs and outputs [51], models can seem like "black boxes" [33,50] to consumers unless steps are made explicit and transparent. This was the rationale for explicitly reporting the steps we took in structuring our model.

In the primary prevention of asthma in children, several studies [8-11,14-18] have provided information on effectiveness but none could singly determine whether primary prevention of asthma is cost-effective and whether a unifaceted or a multifaceted approach is better. Like others [52,53], we have found that the literature is surprisingly silent regarding health economic studies on the value of health care strategies other than those concerned with asthma control. Consequently, one may cast a wary eye on the policy and care decisions relating to the prevention of asthma in children and questions whether the decisions were adequately informed. By synthesising the best available evidence through this model, we hope to take the first step toward properly evaluating and substantiating the choice amongst primary asthma prevention alternatives.

A number of structures could have been used in developing an economic model from regression equations to system dynamic models [21-24]. Some of the more common structures include decision trees, Markov-types, and discrete event simulations (DES) [21-24]. It has always been advocated that the choice of a model structure should be on the consideration of the simplest, and thus best, fit [34] between three elements: the decision problem, natural history of the disease, and current paradigms of care [33]. Since Markov-type models are used in decision problems with time-dependent or recurrent situations [21-24], these were considered inappropriate for this study primarily because asthma was assumed to be a stable diagnosis. Although asthma is understood to be a chronic condition, we considered that its diagnosis would not change in the six-year time frame of our study. This consideration is supported by findings that physiologic changes occur even for so-called mild cases who are more likely to be asymptomatic or only rarely symptomatic [54]. The various wheezing phenotypes could have been modelled instead as the recurring disease states of asthma because of their strong relationship with each other. Despite this, the experts did not think that wheezing was strictly causative of asthma. DES structures, which are more detailed and complicated extensions of Markov-type models [21-24], are similarly not appropriate for this study because asthma does not have a universally accepted measure of severity particularly in population-based studies [55]. Also, the choice for a more complex model should be balanced by any change to the results compared to use of a simpler model [33]. For these reasons, in combination with the decision framework, a decision tree structure was chosen and developed for our specific research question that aims to assess the cost-effectiveness of a programme to prevent the presence of asthma in children around age six years.

The current cost-effectiveness analysis limited itself to the use of the clinical data from the PREVASC studies to demonstrate the model's operation and evaluate its internal validity. The results of this demonstration showed that based on the probability of developing asthma, the current situation dominated either approach to primary prevention of asthma in children. It is notable though that a multifaceted approach was better than a unifaceted one. Use of the PREVASC data enabled technical validation by debugging and refining the model to handle problems like empty cells. For this feasibility assessment of the model, it should be noted that originally, there was no distinction made between the unifaceted and multifaceted groups in the PREVASC studies. The distinction was made only between control and intervention.

### Value of expert panels

Experts are frequently involved in modelling studies to provide the initial model structure, validate the final structure, estimate values of transition probabilities, usage, and cost units [29]. Their reported involvement is, however, uneven [25]. Many are unclear in their reports even of the extent of expert participation in the process. In this paper, we have presented a detailed account of the explicit inputs from experts and their role in structuring a model. We have taken a naïve approach to reduce the bias of the modeller in the selection of one model structure over another but more importantly to benefit from the multi-disciplinary interaction between experts. The round-table discussions provided the opportunity for a dynamic engagement of the experts critical to framing the decision problem. Through the multidisciplinary experts, we hope to have gained better insight into daily practice which is not always possible through systematic reviews alone. All of these should contribute to the improvement of the descriptive and eventual face validity of the model's structure through the iterative reflection on the elicited assumptions. An important consideration is the selection of experts since the breadth and depth of their (practical) knowledge would determine the richness of the endeavour. This could be aided by a brief review of the literature to identify potential experts who are often cited.

Eliciting information and feedback from a panel of experts may range from informal consultations, to round-table discussions, and then Delphi approaches [25,29]. The first approach simply and usually provides background information which may or may not be used. It is typically unilateral and high-handed. Round-table discussions are usually semi-structured sessions where participants are less restricted to explore issues and arguments without necessarily concluding in a consensus which is in contrast to the Delphi approaches. Although Delphi approaches may be seen as the most "democratic" approach to elicit expert opinion because of the intermediary, it is also a handicap since some element of exploration is lost as statements or opinions are "edited" by the intermediary. In round-table discussions, model developers may benefit from the dynamic and personal interaction with and amongst the experts so that no significantly relevant opinion, alternative, or scenario is left out of the model. There is, however, a caveat to round-table discussions: more dominant experts may "drown" the opinions of other experts so the modeller must be able to deftly control the panel. We have tried to address this partly through the imposition of a time limit for the discussions. Also, contributions of each expert were actively sought.

### Parallel validation

Given that novel models do not have comparable models on the same decision problem, the standard validation techniques on the face, convergent, and predictive attributes of this model could not be ascertained at this stage of our study. The comparison of the outputs between the developed model and other models on the same decision problem [33-35] usually covered in the discussion section of a scientific report [56] hints at the prevalent approach to validate novel models. Indeed, validation of novel models "is superseded by the concept of model credibility" particularly because complete validation of a model is not possible [57]. Face validity through expert opinion has been suggested as an approach to validate the structure of novel models. Another solution that we propose and have described here may be through drawing parallelisms with other models, which is to perform what we refer as "parallel validation". Similar to convergent validation in testing between model corroboration [33-35], the distinguishing feature of parallel validation stems from the absence of a model built around the same decision problem. The proposed validation compares other models with the same structure but concern different diseases, populations, or interventions. This is therefore a qualitative consideration of the parallelisms from one model to another. Structural comparisons are admittedly more difficult than comparing input parameters [58]. Nevertheless, decision makers still need to make judicious choices with the highest possible level of confidence. Existing standards on modelling and reporting have been used to serve as markers of similarity and dissimilarity [59]. Figuratively, one can speak of models on the same decision problems as coming from one nuclear family. Parallel validation may be a viable way to compare the structure of one model to a model from a different but closely related family, even if only qualitatively. How modest the literature on (cost-) effectiveness of pharmacotherapies may be, there is even less on prevention [52-54]. Parallel validation reveals that more needs to be done to increase the literature on economic evaluation of primary prevention of asthma amongst children.

## Conclusions

In this paper we showed that a decision-tree structure developed with experts in round-table discussions is descriptively valid in reflecting reality. In addition it is able to incorporate the probable scenarios that would help determine the value of primary asthma prevention. By explicitly detailing the steps in the development of a model from initial problem to final structure, the transparency and use of models for economic evaluation may increase and enrich the toolbox of decision-makers who need to take informed choices. Future studies could determine the number and method of panel sessions that would increase a model's structural validity.

## Competing interests

The authors declare that they have no competing interests.

## Authors' contributions

GFPR wrote the draft of the manuscript, collated and analysed the data as well as reported the results. JLS was involved in all aspects of the study and advised the project group with regard to the economic evaluation. ED was also involved in all aspects of the study, gave advice on effects and process evaluation, and was one of the experts consulted. ADIvA was involved in supervising the technical operation of the model. TRJS and WJCdG were involved in coordinating the data collection as well as writing the manuscript. SK was also involved in writing the manuscript and together with JAK and chief grant applicant OCPvS were responsible for conceptualizing, planning, and supervising the study. All authors read and approved the final manuscript.

## Pre-publication history

The pre-publication history for this paper can be accessed here:

http://www.biomedcentral.com/1471-2288/11/150/prepub

## Supplementary Material

Additional file 1 **Protocol Expert Panel Sessions**. A copy of the protocol to conduct the expert panel sessions. Click here for file
