# Supplementary material for: Structuring and validating a cost-effectiveness model of primary asthma prevention amongst children
Source: BMC Med Res Methodol. 2011 Nov 9;11:150. doi: 10.1186/1471-2288-11-150 (PMC3226537; doi:10.1186/1471-2288-11-150)
Supplement: Additional file 1 — Protocol Expert Panel Sessions. A copy of the protocol to conduct the expert panel sessions. [file 1471-2288-11-150-S1.DOCX]

**INTRODUCTION**

Modelling is the process of structurally synthesising the available data from both primary and secondary sources when full-blown, head-to-head, prospective trials would take years and be logistically as well as financially prohibitive. It also attempts to capture the most salient features of reality by simplifying relationships among important elements of a system being studied. Within the framework of the PREVention of Asthma in Children (PREVASC) research programme, we propose to model the costs and consequences of childhood asthma prevention and a risk-stratification scheme to screen potential prevention candidates. With this model, we hope to be able to provide insight at the hitherto uncertain value of preventing asthma in children as well as the risk-stratification strategy.

For this modelling study, an expert panel will need to be assembled to inform the process of structuring a model as well as verify whether any resulting model is adherent to the state-of-knowledge about asthma prevention. There would need to be at least two each of general physicians and (paediatric) pulmonologists who would provide the necessary bio-clinical knowledge, as well as one health technology assessor who would give guidance on the parameterisation and structuring of the model. Two meetings of at least one hour each would be needed. The meetings are scheduled 29 April and 18 May 2010.

**FIRST MEETING**

**Input**

The first meeting will be prefaced by the framework of prevention (see Figure 1) and an overview of the PREVASC research line which shows the schematic relationship of the relevant studies (see Figure 2). This will be followed by declaring the aim of the research in general and the objective of this study in particular. Finally, the decision problem and perspective as well as assumption-generating questions will be shown.


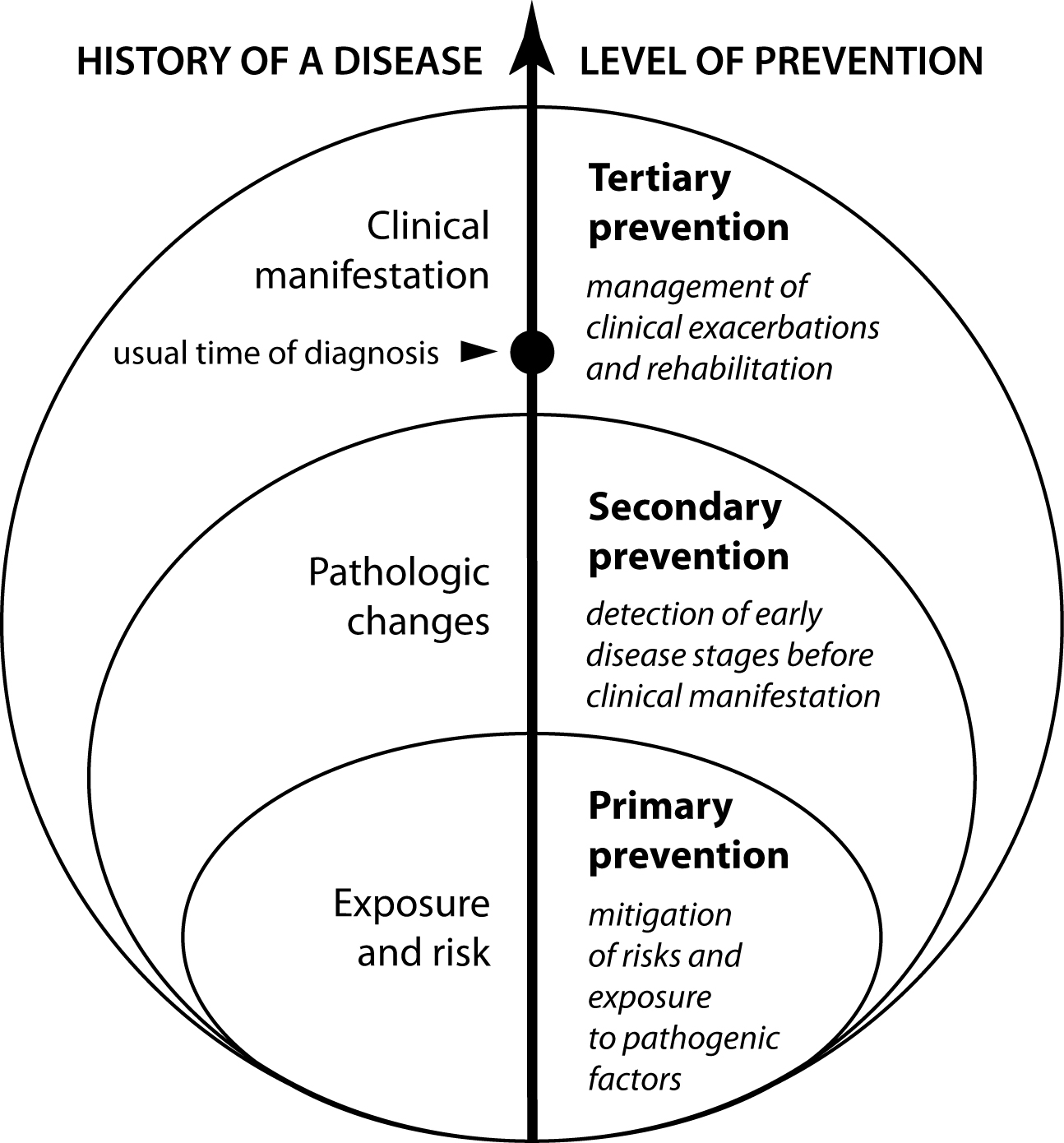


**FIGURE 1. FRAMEWORK OF DISEASE AND PREVENTION**


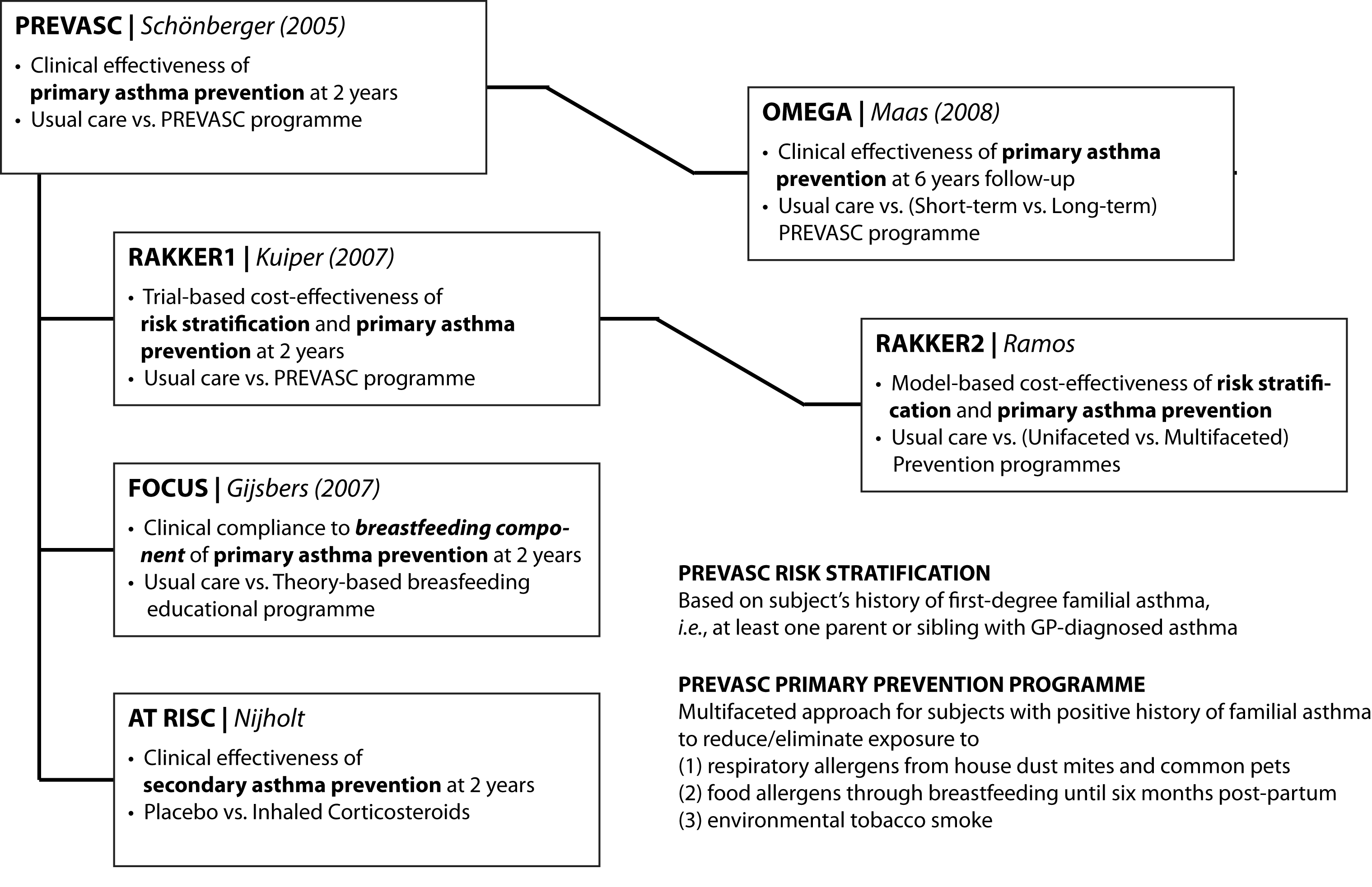


**FIGURE 2. PREVASC RESEARCH LINE**

**AIM**: To determine the economic value of [1] primary prevention of asthma in children and [2] first-degree familial asthma risk stratification.

**OBJECTIVE**: To develop a decision-analytic model to compare the costs and consequences of [1] primary prevention (unifaceted vs. multifaceted) vs. usual care in asthma among children and [2] risk stratification based on the history of first-degree familial asthma, *i.e.*, GP diagnosis of asthma in one parent or sibling. A multifaceted approach, unlike the unifaceted approach, simultaneously employs various prevention actions.

**DECISION PROBLEMS AND PERSPECTIVE**: At the primary care level, what is the best approach to the prevention of asthma in children and how can the target individuals be best identified?

**ASSUMPTION-GENERATING QUESTIONS**:

1. What is the state-of-knowledge about the pathogenesis of asthma? How is this related to the levels of prevention?
2. Is there a gold standard for the effects of asthma care that are feasibly measurable in the GP surgery? Are there alternatives and how might these be measured?
3. Is it important to analyse the outcomes at the individual or aggregate level?
4. Are there asthma-related comorbidities that might affect the outcomes?
5. What is the clinically meaningful length of time before the different approaches to asthma prevention become appreciable?
6. How can risk for asthma be feasibly determined in the GP surgery and how can it be used within prevention programmes?
7. Are there individual risk factors that might affect the outcome in a non-linear fashion?

**Process**

The figure of the levels of prevention in relation to the general evolution of a disease would be used as a reviewer. The PREVASC research line would be used to provide a historical context to the current study and the research programme in which it is embedded. It should also provide insight into the currently available data that could be used in the eventual model simulation. The aim, objective, decision problems and decision context would also help focus and delineate the discussions that would arise from the assumption-generating questions. The questions themselves are open-ended so to help focus the discussions, at least five minutes would be devoted to each question. More or less time would be devoted to other questions subject to need and availability of time.

**Output**

A roster of assumptions dealing with the natural course of the disease, comparators, measurement outcomes, and time horizon, among others, should result from the discussions of the experts.

**INTERIM**

It should be apparent from the assumptions what the elements and structure of the model might be. At this point a (rough) sketch of the model structure can already be done.^[[1]](#footnote-1)^ Once a reasonable structure has been developed in accordance with the assumptions, a systematic search of the published literature should be performed to determine whether a model structure already exists similar to the one being developed. For this modelling study, the scheme of systematic search is shown in Figure 3. Surveying the literature at this stage for a model that fits the assumptions after a model is built is not an exercise on the reinvention of the wheel because it would serve to validate the structure of the model (parallel validity). Even when found models may be better than the developed one, the differences would also point to valuable learning moments on model building. The differences might even point to relevant considerations or innovative approaches to existing problems that could be presented as input to the next expert meeting.


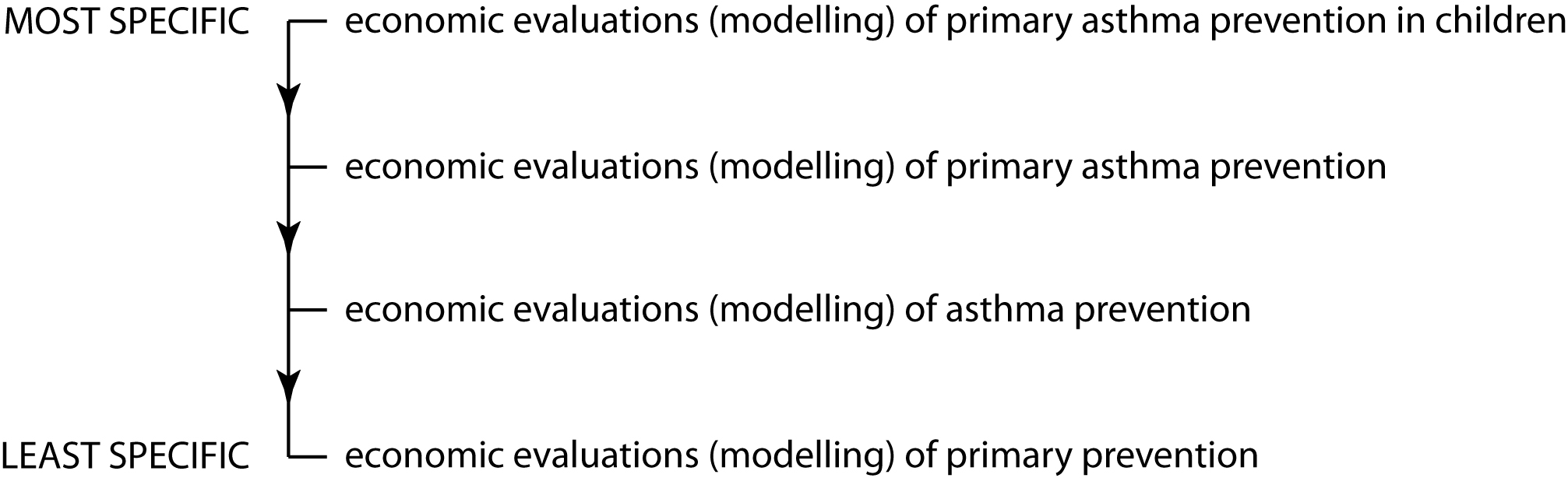


**FIGURE 3. SYSTEMATIC SEARCH STRATEGY SCHEME**

**SECOND MEETING**

**Input**

The second meeting will be prefaced by a brief overview of the modelling process (see Figure 4) and the general types of economic models with their most distinctive differences. This step is taken here so as not to colour and limit the generation of assumptions determined during the first meeting by a preconception of the final model structure. With this in mind, the (found or developed) model structure would be presented to the experts.


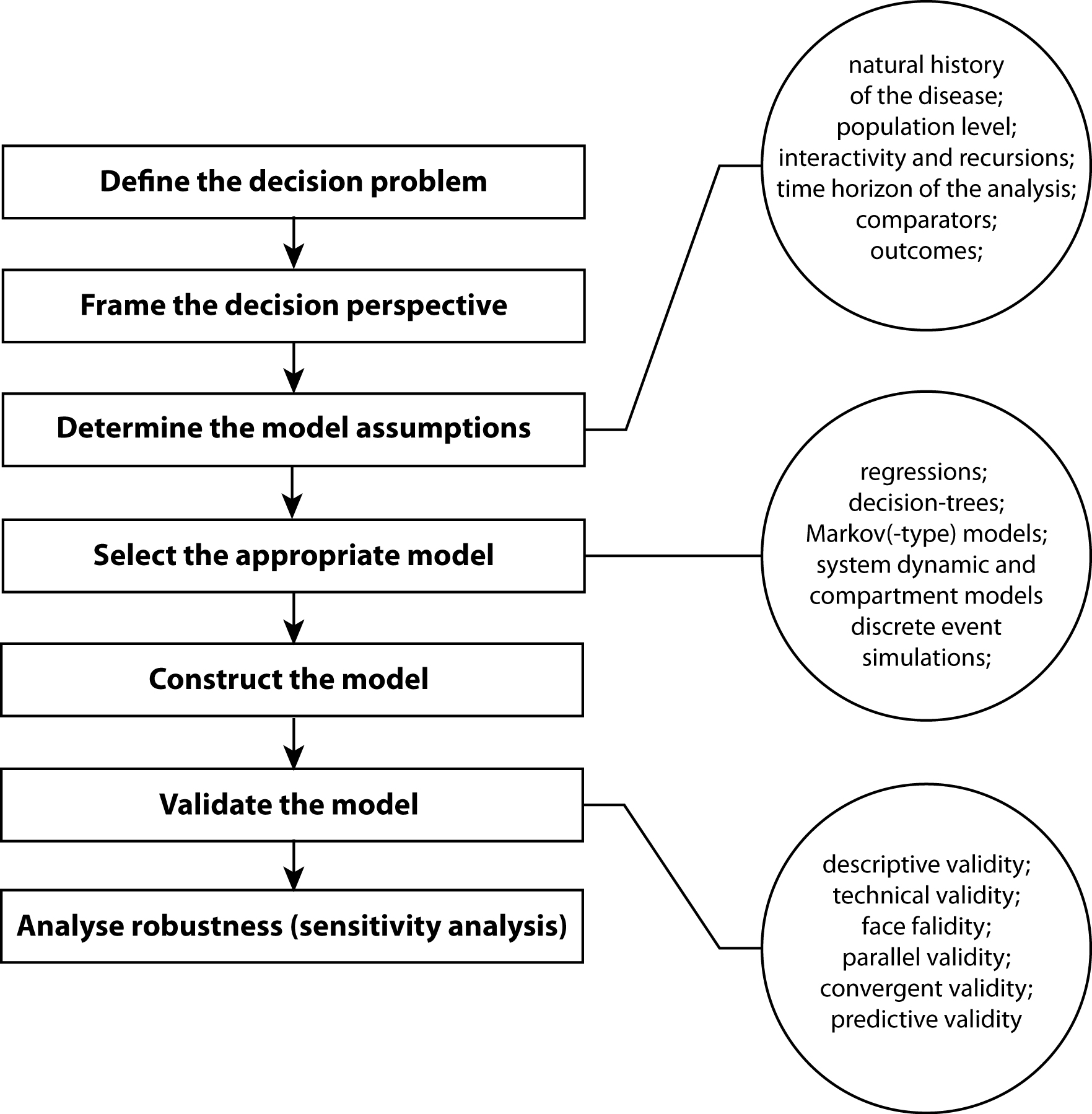


**FIGURE 4. MODELLING PROCESS**

**Process**

Assessment of how the model structure reflects the assumptions made during the first meeting would be done using questions covering seven themes from the checklist drawn up by Soto.^[[2]](#footnote-2)^ These themes are checked using the following questions:

**HYPOTHESIS AND OBJECTIVE**

1. Is the hypothesis stated in a testable form?
2. Does a well-defined objective exist? Is it clear, explicit and answerable?

**RATIONALE**

1. Is the rationale of the model properly stated?
2. Is the need for modelling vs. alternative methodologies discussed?

**TYPE AND DESCRIPTION OF MODEL**

1. Is the type of model the most appropriate according to the objective sought and the hypothesis?
2. Is the design of the model appropriate when considering the literature review, previous models performed, and research questions?
3. Does the model represent the daily medical practice in the country where it was built?
4. Is the description of the model complete enough to allow replication?

**TIME HORIZON**

1. Is the time horizon selected appropriate in accordance with the hypothesis and objective of the study?
2. Will alternatives be able to show all outcomes with this time horizon?

**PERSPECTIVE**

1. Is the perspective of the study clearly specified?
2. Is it justified properly according to the questions and objectives?

**ASSESSMENT AND COMPARATORS**

1. Are all relevant treatment alternatives considered and described in sufficient detail?
2. Is the rationale for choosing the alternatives compared stated?
3. Are any important comparators omitted? Is (should) a do-nothing alternative (be) considered?

**OUTCOMES AND PROBABILITIES ASSESSMENT**

1. Are the main outcomes measured in the analysis? Are they the most relevant?
2. Is the outcome selected the most relevant for the study (clinical measures, health endpoints, life-years gained, QALYs, deaths avoided)?

Based on the guiding questions at this meeting, the found model (if any) would be compared to the developed model to see which best reflects the assumptions made in the first meeting. It would also be the opportunity to inform the refinement of the model structure, whether it is the found or developed one.

**Output**

At the end of the second meeting, the experts should have made a choice between the found or developed model and have pointed out how the model would look in its final form. This means the product is a figure or a schematic representation.

1. Varian HR. How to build an economic model in your spare time [version 25 July 2009]. In; Szenberg M (editor). *Passion and Craft: Economists at Work.* Michigan: University of Michigan Press; 1997. Downloaded from http://people.ischool.berkeley.edu/~hal/Papers/how.pdf [↑](#footnote-ref-1)
2. Soto J. Health economic evaluations using decision analytic modeling: Principles and practices—utilization of a checklist to their development and appraisal. *Int J Technol Assess Health Care* 2002;18(1):94-111. [↑](#footnote-ref-2)
